# A Greek Real-World Experience With the Implementation of the EndoPredict Gene Signature in a Cohort of Pre-menopausal and Post-menopausal Patients With ER+/HER2- Breast Cancer

**DOI:** 10.7759/cureus.114838

**Published:** 2026-08-20

**Authors:** George Pantazidis, Antigoni Sourla, Aggeliki Vasiageorgi, Christina Yfanti, Anna-Maria Alisandrou, Giasemi Stefanou, Aggelos Petsavas, Fotini Pavlidou, Styliani Parpoudi, Dimitrios Alexandrou, Anna Sachoulidou, Annezo Marinatou, Evgenia Kalogridaki, Theodoros Kontoulis, Panagiotis E Daskalakis, Vlassios Tsantilas, Stavros Chatzopoulos, Christos Kanistras, Theodoros Argyriou, Dimitrios Dragoumis, Vaia Stafyla, Konstantinos Louis, Andriana Kouloura, Ioanna Galanou, Georgios Papazoglou, Dimitrios Grosomanidis, Konstantinos Chatzopoulos, Georgia Pasaskevakou, Panagiota Varnava, Anna Giannopoulou, Xenofon Xenakis, Apostolos Mpakogiannis, Ioannis Flessas, Dimitrios Nasikas, Dimitrios Koronarchis, Stergios Douvetzemis, Ioannis Fyssas, Christos Spanopoulos, Dimosthenis Baltas, Dimitrios Keramopoulos, Konstantinos Bouloukos, Vilma Gaki, Dionysios T Dimas, Ioannis L Missitzis, Georgios Rigas, Christos Kalyvopoulos, Stavros Intzes, Stefanos Zervoudis, Europi Michailidou, Anastasios Katsourakis, Markos Thanos, Apostolos Zavos, George Boutsikos, Maria Kanara, Sotirios Α Rousogiannis, Panagiotis Charatsaris, Pavlos Lampropoulos, Sarantos Papadopoulos, Evangelos P Solakis, Ioannis Askoxylakis, Sofia Kakoulaki, Eleftherios Sfakianakis, Nikolaos Vrentzos, Despoina Milonaki, Ioannis Kyriasanos, Foivos Irakleidis, Rodoniki Iosifidou

**Affiliations:** 1 Genetics and Molecular Biology, Bioiatriki Healthcare Group, Athens, GRC; 2 Anatomical Pathology, Bioiatriki Healthcare Group, Athens, GRC; 3 Surgical Oncology, Theagenio Cancer Hospital, Thessaloniki, GRC; 4 Surgical Oncology, School of Medicine, Aristotle University of Thessaloniki, Thessaloniki, GRC; 5 Breast Surgery, Ηelena Venizelou General and Maternity Hospital, Athens, GRC; 6 Surgical Oncology, Genesis Hospital, Thessaloniki, GRC; 7 Surgical Oncology, Bioclinic Thessaloniki, Thessaloniki, GRC; 8 Surgical Oncology, St. Luke's Hospital, Thessaloniki, GRC; 9 4th Department of Surgery, Attikon University Hospital, National and Kapodistrian University of Athens, Athens, GRC; 10 3rd Department of Obstetrics and Gynecology, Attikon University Hospital, National and Kapodistrian University of Athens, Athens, GRC; 11 Surgical Oncology, Mitera Hospital, Athens, GRC; 12 Surgical Oncology, Henry Dunant Hospital Center, Athens, GRC; 13 Surgical Oncology, Metropolitan General Hospital, Athens, GRC; 14 Gynecologic Oncology, Rea Maternity Hospital, Athens, GRC; 15 Gynecologic Oncology, IASO Hospital, Athens, GRC; 16 Surgical Oncology, IASO Hospital, Athens, GRC; 17 Breast Unit, Athens Medical Center, Psychiko Clinic, Athens, GRC; 18 Breast Surgery, Agios Savvas Athens General Cancer and Oncology Hospital, Athens, GRC; 19 Surgical Oncology, Interbalkan Medical Center, Thessaloniki, GRC; 20 Obstetrics and Gynaecology, Rea Maternity Hospital, Athens, GRC; 21 General Surgery, Agios Pavlos General Hospital, Thessaloniki, GRC; 22 Surgical Department, General Hospital of Thessaloniki "o Agios Dimitrios", Thessaloniki, GRC; 23 Gynecologic Oncology, Ygeia Hospital, Larissa, GRC; 24 Gynecologic Oncology, University of Thessaly, Larissa, GRC; 25 Surgical Oncology, IASO Thessalias, Larissa, GRC; 26 Surgical Oncology, General Hospital of Trikala, Trikala, GRC; 27 Surgery, General Hospital of Volos, Volos, GRC; 28 Surgical Oncology, University Hospital of Ioannina, Ioannina, GRC; 29 Breast Unit, Metaxa Cancer Hospital, Athens, GRC; 30 Gynecologic Oncology, Blue Cross Clinic, Athens, GRC; 31 General Surgery, 401 General Military Hospital of Athens, Athens, GRC; 32 Surgical Oncology, Iasis Gavrilakis SA General Clinic, Chania, GRC; 33 Surgical Oncology, Asklepieion Creta Therapeutic and Diagnostic Center, Heraklion, GRC; 34 Surgical Oncology, Creta Interclinic, Heraklion, GRC; 35 Surgical Oncology, General Hospital of Heraklion Venizeleio-Pananeio, Heraklio, GRC; 36 General Surgery, General Hospital of Nikaia "Agios Panteleimon", Athens, GRC; 37 Surgery, Naval and Veterans Hospital of Athens, Athens, GRC; 38 Surgical Oncology, Rhodes General Clinic, Rhodes, GRC

**Keywords:** breast cancer, endopredict, epclin risk score, gene signature, pre and post-menopausal

## Abstract

Background

EndoPredict® is a guideline-recommended gene signature assay guiding whether adjuvant treatment should include chemotherapy or not. The EndoPredict® Clinical (EPclin) Risk Score combines molecular and clinical data to predict the distant recurrence of breast cancer, providing valuable information about the prognosis and chemotherapy benefit in both pre- and post-menopausal patients.

Methodology

This is a real-world, retrospective study aiming to characterize the EPclin Risk Score’s utility in 200 consecutively selected Greek patients with estrogen receptor positive/human epidermal growth factor receptor 2 negative (ER+/HER2-) breast cancer, enrolled in a single institution, considering their clinicopathological features and menopausal status, with emphasis on its role in identifying patients suitable for treatment de-escalation.

Results

In this study, 100 pre-menopausal and 100 post-menopausal ER+/HER2- breast cancer patients were included. Among the pre-menopausal subgroup, 65% of the patients were classified as low risk and the other 35% as high risk, while among the post-menopausal subgroup, the low-risk and high-risk patients were 66% and 34%, respectively. A notable 30% of node-positive premenopausal patients were characterized by EPclin as at a low risk of breast cancer.

Conclusions

The EPclin Risk score has the ability to stratify and guide therapy decisions in ER+/HER2- breast cancer patients. It classifies the recurrence risk differently from traditional assessments and identifies low-risk premenopausal patients with node-positive disease. This may provide a de-escalation treatment pathway for such patients but requires further clinical validation.

## Introduction

Breast cancer is the most commonly diagnosed malignancy worldwide and remains a leading cause of cancer-related mortality. According to the World Health Organization (WHO, 2020) [1], it ranks as the fifth leading cause of cancer death globally, while more recent data indicate that it is the second most frequently diagnosed cancer and the fourth leading cause of cancer-related death worldwide (WHO, 2022) [2].

Breast cancer is a heterogeneous disease classified according to the molecular expression of hormone receptors (HR), namely estrogen receptor (ER) and progesterone receptor (PR), as well as the human epidermal growth factor receptor 2 (HER2). Based on these biomarkers, tumors are categorized as the following subtypes: HR-positive/HER2-negative, HER2-positive, and triple-negative (HR-negative/HER2-negative). Among these, patients with HR-positive/HER2-negative tumors (invasive breast cancer) generally demonstrate the best prognosis, followed by those with HER2-positive disease, while triple-negative breast cancer is associated with the least favorable outcomes [3].

Early ER-positive, HER2-negative tumors are a heterogeneous group of tumors with variable prognosis. Although patients with this type of tumor derive substantial benefit from endocrine therapy, only a subset experience clinically meaningful improvement with adjuvant chemotherapy [4]. Consequently, an accurate risk stratification is essential to optimize treatment selection and avoid unnecessary toxicity for patients with localized ER+/HER2- breast cancer.

A number of commercially available prognostic and predictive assays are routinely used in clinical practice to inform the risk of recurrence and the benefit of chemotherapy for such patients. Their performance may differ depending on the patient's menopausal status [5]. While their clinical utility is well established in node-negative and post-menopausal patients, their value in pre-menopausal, node-positive breast cancer remains a controversial field in molecular oncology. Recent prospective randomized studies, including the Oncotype DX TAILORx (Trial Assigning Individualized Options for Treatment (Rx)), RxPonder trials, and the Mammaprint MINDACT (Microarray In Node negative Disease may Avoid ChemoTherapy) trial, have shown that molecular signatures in women differ in their prognostic ability and predictive power based on their menopausal status. The findings of these trials also suggest that the benefit of chemotherapy in pre-menopausal women may be related to chemotherapy-induced amenorrhea, which could potentially be achieved with ovarian function suppression (OFS). Ongoing trials, such as the OPTIMA (Optimal Personalised Treatment of early breast cancer using Multi-parameter Analysis) trial and the OFSET (Evaluating the Addition of Adjuvant Chemotherapy to Ovarian Function Suppression Plus Endocrine Therapy in Premenopausal Patients With pN0-1, ER-Positive/​HER2-Negative Breast Cancer and an Oncotype Recurrence Score Less Than or Equal to 25) study, which include OFS as part of the endocrine therapy in all pre-menopausal women, may clarify this question [6].

On the contrary, EndoPredict® (Myriad Genetics, Salt Lake City, UT) has been prognostic of 10-year distant recurrence in pre-menopausal women, independently of nodal status [5]. Overall, EndoPredict® is a validated multigene expression assay that identifies a substantial proportion of patients (approximately 55%-65% in clinical trials) with an excellent long-term prognosis who can safely forgo adjuvant chemotherapy. By integrating molecular and clinical parameters, EndoPredict® provides robust prognostic information regarding distant recurrence risk and predictive insight into chemotherapy benefit [7-10].

The test is supported by Level 1A evidence and is recommended in multiple national and international clinical practice guidelines, including those from the European Society for Medical Oncology [11-15]. Its use enables more precise, individualized treatment decisions by accurately predicting distant recurrence risk [7,16] and chemotherapy benefit [17]. Thus, it reduces both overtreatment and undertreatment, minimizing unnecessary adverse effects and improving patients’ quality of life.

The EndoPredict® Clinical (EPclin) Risk Score combines the 12-gene molecular score with key clinical variables, specifically tumor size and nodal status, and has been demonstrated to be an independent and clinically relevant predictor of distant recurrence. Importantly, it provides additional prognostic and predictive value beyond standard clinicopathological factors alone [7].

The objective of the present study was to evaluate the utility of the EPclin Risk Score in terms of how risk classification, recurrence estimates, and chemotherapy benefit estimates for a cohort of Greek patients with ER+/HER2- early breast cancer are contextualized to their clinicopathological features and menopausal status. The EPclin score’s potential to identify patients suitable for treatment de-escalation has been a key focus.

## Materials and methods

Study cohort

Samples from a total of 200 women with breast cancer were included in this study. This comprised a subgroup of 100 pre-menopausal and a subgroup of 100 post-menopausal patients who were tested consecutively from April 20, 2026, backward using the EPclin gene signature assay in a single laboratory until the desired number of 100 for each subgroup was reached.

Samples were eligible for inclusion in the study if the patient was of age >18 years, had a diagnosis of localized ER+/HER2- breast cancer, had the availability of a conclusive EPclin assessment, and had a date of EPclin testing prior to April 20, 2026. No exclusion criteria were applied to ensure the absence of selection bias.

Methods

Tumors were staged according to the TNM (tumor, node, metastasis) classification system, as defined by the American Joint Committee on Cancer and the Union for International Cancer Control [18].

Formalin-fixed, paraffin-embedded (FFPE) tumor tissue samples were sectioned at 5-10 μm thickness, depending on pathological assessment of tumor cellularity. Only samples with >30% tumor cell content were included, in accordance with the established guidelines.

RNA was extracted using the Maxwell® RSC RNA FFPE kit (Promega Corporation, Madison, WI). Following quality control (QC) assessment of RNA quantity and integrity, aliquots were used for EndoPredict® analysis.

Gene expression was quantified in triplicate using reverse transcription quantitative real-time PCR (RT-qPCR). The assay measures eight cancer-related genes (AZGP1, BIRC5, DHCR7, IL6ST, MGP, RBBP8, STC2, UBE2C), three reference genes (CALM2, OAZ1, RPL37A), and one control gene to detect residual genomic DNA. Reactions were performed using EndoPredict® reagents (Myriad Genetics/Eurobio (Eurobio Scientific Service GmbH, Rheinbach-Allemagne, Germany)) on a QuantStudio™ 5 Dx Real-Time PCR system (Applied Biosystems, Waltham, MA).

Raw RT-qPCR data were exported as .txt files and analyzed using the web-based EndoPredict® Report Generator (EPRG). The 12-gene molecular score (EPscore) was calculated from normalized gene expression values and subsequently combined with tumor size and nodal status to generate the EPclin Risk Score. The EPclin Risk Score provides: (i) 10-year risk of distant recurrence with endocrine therapy alone, (ii) risk of late recurrence (years five to 15) in patients free of distant recurrence after five years of endocrine therapy alone, and (iii) estimated absolute chemotherapy effect at 10 years [7,9].

Testing parameters

Both the 12-Gene Molecular Score and EPclin Risk Score were developed and trained in a multicenter cohort of 964 treatment-naive FFPE primary breast cancer samples [16]. A Cox proportional hazards model was used to derive the EPclin Risk Score as a linear combination of the molecular score, tumor size, and nodal status. According to the model, the EPclin Risk Score is calculated as:

$\mathrm{EPclin\ Risk\ Score} = (0.35 \times \mathrm{Tumor\ Size}) + (0.64 \times \mathrm{Nodal\ Status}) + (0.28 \times \mathrm{EPscore})$ [7,9].

Tumor size was categorized as: T1ab (≤1 cm), T1c (>1 but ≤2 cm), T2 (>2 but ≤5 cm), and T3 (>5 cm). Nodal status was categorized as: N0 (node-negative), N1 (one to three positive lymph nodes or micrometastases), and N2 (four to 10 positive lymph nodes).

Both the EPscore and EPclin Risk Score have been validated in multiple independent cohorts [7,9].

Interpretation of EPclin Risk Score

EPclin Risk Scores were interpreted according to predefined thresholds [7,9]:

Low risk (EPclin: 1.0-3.3): associated with a low 10-year risk of distant recurrence. This report states first the estimated 10-year risk of recurrence; second, the likelihood of distant recurrence five to 15 years after diagnosis; and finally, the estimated absolute benefit of chemotherapy at 10 years.

High risk (EPclin: 3.4-6.0): associated with a high 10-year risk of distant recurrence. This report includes the same results as in the low risk.

Scores outside the validated range: Scores >6.0 (up to 8.2) are reported as high risk; however, quantitative estimates for recurrence risk and chemotherapy effect are not provided. Scores <1.0 are not reportable.

Statistics

This was a non-interventional study and therefore epidemiological methods were applied in the analysis, and no formal hypotheses were tested. Descriptive statistical analysis was performed for all study data. Continuous variables were summarized with the use of descriptive statistical measures (mean value, median value, minimum value and maximum value). Categorical/distinct variables were displayed as frequency tables (N, %).

Data collection and availability

All EPclin data were generated and collected in the laboratory of Bioiatriki S.A. All clinical and pathological data were collected from the pathology reports accompanying each patient sample included in the study. Only the information relevant to the study objectives was collected, completely anonymized.

The data generated in this study are available upon reasonable request from the corresponding author.

Ethical oversight

The Scientific Commission of Bioiatriki S.A. approved the protocol and granted a waiver of informed consent provided that all personal patient data were effectively anonymized in accordance with applicable laws and national regulations.

Disclaimer: The present study had no clinical implications and patient-related decisions were made routinely by the treating physicians and were not influenced by the study’s results.

## Results

This retrospective study analyzed data from a Greek cohort of 200 patients who underwent EndoPredict® testing between April 20, 2025 and April 20, 2026 at the Molecular Biology Laboratory of Bioiatriki S.A.

The baseline characteristics of the study cohort, including tumor size, nodal status, grade, and the distribution of Ki-67 expression are depicted in Table 1. Ki-67 subgroups were defined as per Constantinidou et al. [5]. Among the pre-menopausal patients (n=100), there were 76 with T1 disease and 24 with T2; 77 were N0 and 23 were N1. Among the post-menopausal patients (n=100), there were 77 with T1 tumor, 22 with T2 and one patient with T3; 83 were N0 and 17 N1.

**Table 1:** Baseline characteristics for the pre-menopausal and the post-menopausal subgroups.

Characteristic	Pre-menopausal	Post-menopausal
Age (y) at diagnosis (mean)	45.0	67.5
Tumor size (TNM stage), N (%)		
pT1ab	30	27
pT1c	46	50
pT2	24	22
pT3	0	1
Nodal Status, N (%)		
Negative (N0)	77	83
Positive (N1)	23	17
Tumor Grade, N (%)		
I	20	16
II	60	67
III	19	13
Inconclusive, I or II	0	2
Inconclusive, II or III	1	2
Ki-67 expression (categorical), N (%)		
≤5%	8	8
6% - 29%	76	73
≥30%	13	16
Intermediate score between above subgroups	3	3

In the pre-menopausal group of patients, 65 subjects (65%) were classified as low risk (EPclin Risk Score <3.3) and 35 subjects (35%) as high risk (EPclin Risk Score >3.3). Respectively, in the post-menopausal group of patients, 66 subjects (66%) were classified as low risk and 34 subjects (34%) as high risk. Categorization per EPclin Risk score and TNM staging is summarized separately for the pre-menopausal and the post-menopausal subgroups in Table 2.

**Table 2:** Categorization of pre- and post-menopausal ER+/HER2- early breast cancer patients according to TNM staging (Tumor size - Nodal status) and EndoPredict® scoring: EPscore <5 to EPscore >5 and EPclin Risk Score <3.3 to EPclin Risk Score >3.3. ER+/HER2-: Estrogen receptor positive/human epidermal growth factor receptor 2 negative.

Tumor size	pT1		pT2		pT3		
Nodal status	N0	N1	N0	N1	N0	N1	
Pre-menopausal	22	4	7	2	-	-	EPscore <5
	38	12	10	5	-	-	EPscore >5
	60	16	17	7			Total (N=100)
	50	7	8	-	-	-	EPclin Risk Score <3.3 (low risk)
	10	9	9	7	-	-	EPclin Risk Score >3.3 (high risk)
Post-menopausal	26	4	6	1	1	-	EP score <5
	39	8	11	4	-	-	EP score >5
	65	12	17	5	1		Total (N=100)
	53	5	7	1	-	-	EPclin Risk Score <3.3 (low risk)
	12	7	10	4	1	-	EPclin Risk Score >3.3 (high risk)

The categorization as per EPclin Risk score and Ki-67 expression subgroups is summarized separately for the pre-menopausal and the post-menopausal subgroups in Table 3.

**Table 3:** Categorization of pre- and post-menopausal ER+/HER2- early breast cancer patients according to Ki-67 expression and EndoPredict® scoring: EPscore<5 to EPscore>5 and EPclin Risk Score<3.3 to EPclin Risk Score>3.3.

	Ki-67			
	≤5%	6-29%	≥30%	
Pre-menopausal	5	28	2	EP score <5
	3	48	14	EP score >5
	8	76	16	Total (N=100)
	7	51	7	EPclin Risk Score <3.3 (low risk)
	1	25	9	EPclin Risk Score >3.3 (high risk)
Post-menopausal	7	30	1	EP score <5
	1	46	15	EP score >5
	8	76	16	Total (N=100)
	8	54	4	EPclin Risk Score <3.3 (low risk)
	-	22	12	EPclin Risk Score >3.3 (high risk)

**Figure 1:**
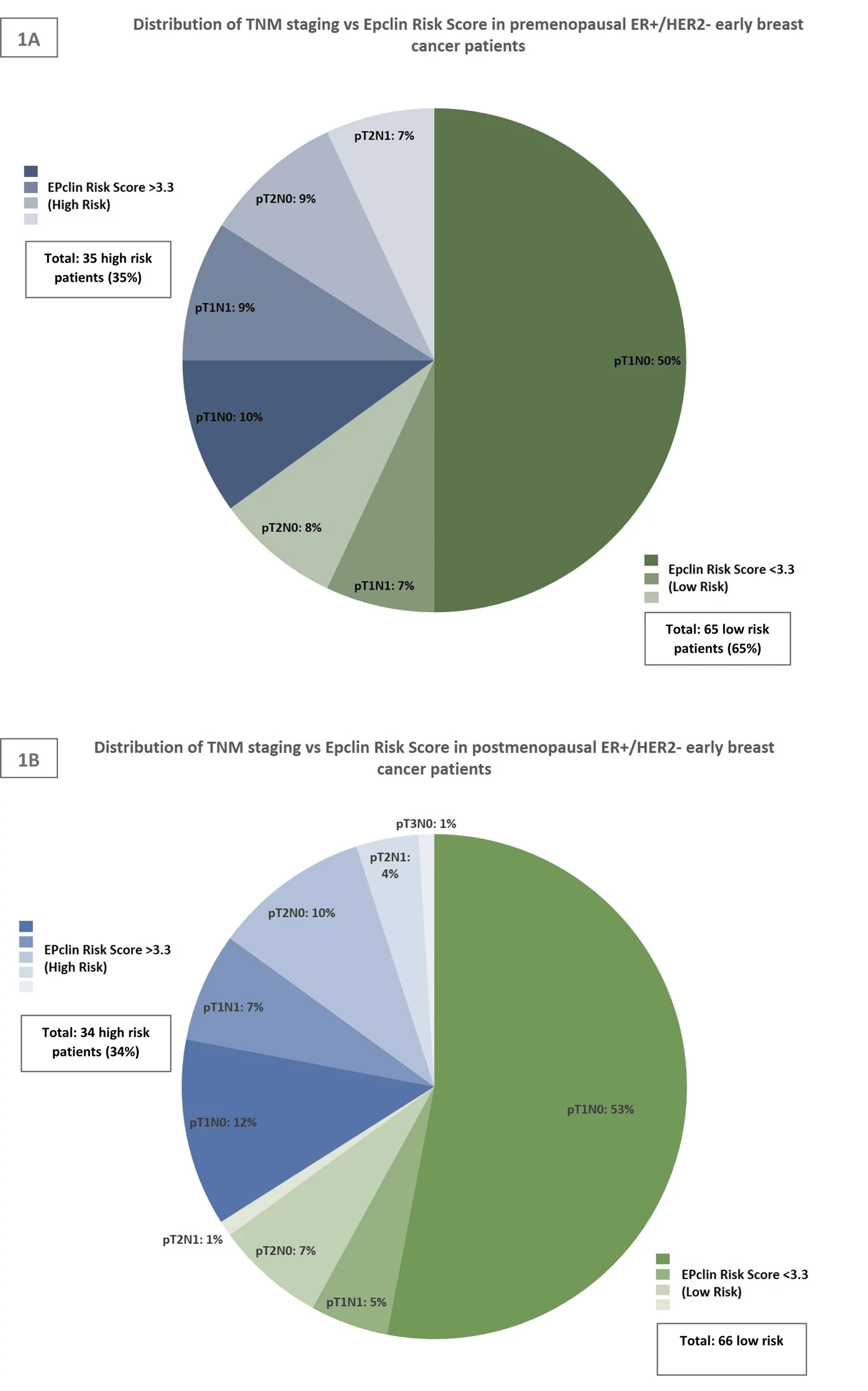
Distribution of tumor size and nodal status vs EPclin Risk Score in 100 pre-menopausal (Panel 1A) and 100 post-menopausal (Panel 1B) ER+/HER2- early diagnosed breast cancer patients.

The distribution of TNM staging versus EPclin Risk Score in the pre-menopausal subgroup is depicted in Figure 1A and the respective distribution in the post-menopausal subgroup in Figure 1B. In the pre-menopausal subgroup, all TNM stages are represented in both high- and low-risk EPclin score groups, with the exception of patients with pT2N1 breast cancer, who were all identified as high risk (no pT3 pre-menopausal patients were available in our analysis). Of note, 7/16 (43.8%) among pre-menopausal patients with pT1N1 disease and 7/23 (30.4%) with node-positive disease overall were identified as having a low risk of recurrence. In the post-menopausal subgroup, all but one patient with pT2N1 disease were identified as patients with high risk of recurrence and the single patient with T3 disease was also reported to have a high risk.

Considering Ki-67 expression and grade and among patients with node-positive disease: in the pre-menopausal subgroup, 2/2 with grade 3 and 10/12 with Ki-67 ≥20% were allocated to EPclin high-risk, and 5/23 had EPclin ≥4.8, whilst in the post-menopausal subgroup, these numbers were 1/1, 3/3 and 2/17, respectively.

**Figure 2:**
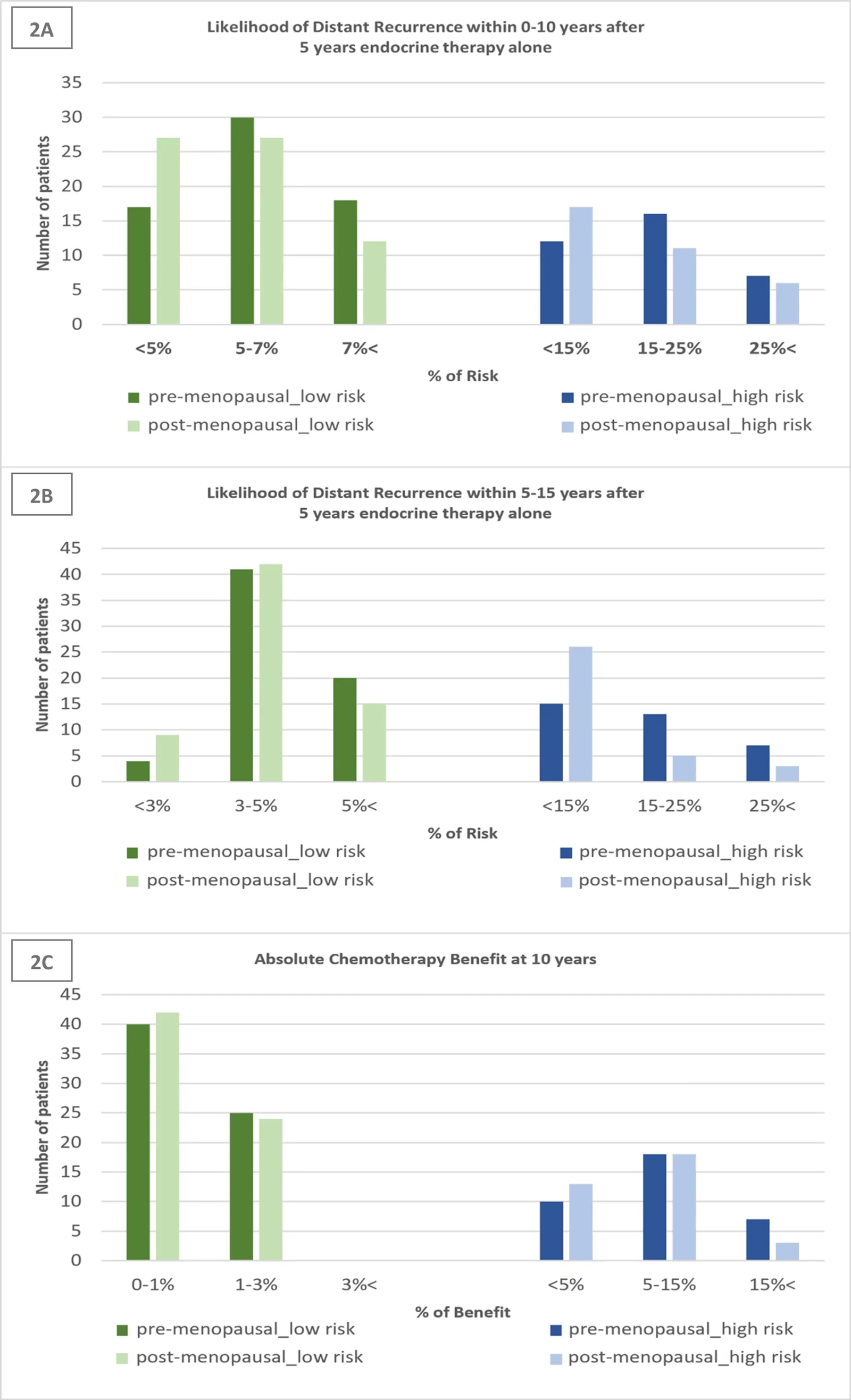
Schematic representation of the likelihood of distant recurrence within 0-10 and 5-15 years after five years of endocrine therapy alone (Panels A and B), as well as the absolute chemotherapy benefit both in pre- and post-menopausal patients (Panel C), in low- and high-risk categories.

The distribution of early and late recurrence risk, as well as estimated chemotherapy benefit, separately in pre-menopausal and post-menopausal patients is shown in Figure 2. Among low-risk patients, both pre-menopausal and post-menopausal groups cluster predominantly within the 5% (17 pre-menopausal and 27 post-menopausal patients) and 5%-7% (30 pre-menopausal and 27 post-menopausal patients) categories for 10-year distant recurrence risk. In terms of the risk of late recurrence (years five to 15), most patients (41 pre- and 42 post-menopausal patients) were allocated within the 3%-5% range, while the estimated absolute chemotherapy benefit is minimal, typically ranging from 0% to 3% (65 pre-menopausal and 66 post-menopausal patients).

Among high-risk patients, the distribution of early and late risks of distal recurrence demonstrates a greater tendency for the pre-menopausal cohort to report higher rates of these risks: Considering early recurrence, in the pre-menopausal cohort, 12 patients were reported to have a risk <15% and 23 patients a risk >15%. In the post-menopausal cohort, 17 patients were reported to have a risk <15% and 17 a risk >15%. Considering late recurrence, more patients in the pre-menopausal cohort were identified with risk >15% (20 out of a total of 35) whilst most post-menopausal patients were allocated to the <15% risk group (26 out of a total of 33). Similarly, a marked difference in the estimated absolute chemotherapy benefit of >15% is observed with more pre-menopausal patients being allocated to that group than the post-menopausal group (seven vs three patients).

## Discussion

Since Greece introduced a systematic breast cancer screening program five years ago, early diagnoses have increased, reducing the number of patients with advanced stages of breast cancer. Gene expression signatures are included in international treatment guidelines to support and optimize decision-making in adjuvant systemic therapy for ER+/HER2- early breast cancer patients. These genomic tools identify patients for whom chemotherapy is beneficial and those with a low risk of recurrence and excellent prognosis who could avoid undergoing chemotherapy [6].

EndoPredict®, a second-generation genetic signature test, combines gene expression levels of 12 genes with tumor size and nodal status to predict the probability of late distant recurrence 0-10 years after diagnosis and five to 15 years after diagnosis for patients free of disease and after five-year endocrine therapy alone. It also estimates the 10-year absolute benefit of chemotherapy.

Overall, EndoPredict allocated 65%/66% in the low-risk group (in the pre-/post-menopausal subgroups) and 35%/34% in the high-risk group (respectively). These rates are similar to the ones reported by Klein et al. [19].

Considering tumor size and nodal status information, the parameters incorporated in the EPclin algorithm, the majority of breast-cancer patients with small tumor size (T1a, T1b, T1c) and negative nodal status (N0) were indeed identified as low risk based on EndoPredict® testing. However, a marked percentage (11%) of both pre- and post-menopausal women, with specific TNM staging classification, were reported with an EPclin Score >3.3 (high risk). This is in line with the main EndoPredict validation report by Filipits et al., where EPclin successfully provided additional prognostic information on the risk of distant recurrence of breast cancer patients, independently of clinicopathologic parameters [7].

Within the same context, EndoPredict® also identified a small percentage of patients (8%) with clinically high-risk characteristics (T2/N1 or T3/N0) who were characterized as low risk. Sestak et al. and Ettl et al. also demonstrated that EndoPredict classified patients with clinically high risk, tumors as low risk (EPclin Score<3.3) who either received unnecessary chemotherapy [17] or could safely forgo the use of chemotherapy [20].

Regarding the pre-menopausal subgroup, the specific low-risk rate (65%) also aligns with the respective rate reported in Constantinidou et al. [5], which only studied pre-menopausal patients. This low-risk subgroup includes patients with node-positive disease, with the present study reporting 30.4% of such patients being characterized as low risk, a rate higher than what Constantinidou et al. reported (19.4%) [5], but this difference may be attributed to the subgroup’s small sample size and the overall retrospective nature of the present study. This study therefore agrees with the previous reports of Constantinidou et al. [5] and Klein et al. [19], according to which EndoPredict has the ability to identify a specific subgroup of pre-menopausal ER+/HER2- early breast cancer patients with node-positive disease as low risk, therefore enabling treating physicians to de-escalate treatment and avoid chemotherapy. Conversely, the absence of follow-up information, including the level of adherence to the guidance of the EPclin score, deprives this study of more robust data compared to the aforementioned international literature.

No differences in the distribution of EPclin likelihoods of recurrence can be concluded between the two study subgroups mainly due to the small sample size of these groups. In the low-risk category, no considerable differences between the pre- and post-menopausal group were observed. More notable numerical differences were observed in the high-risk group, where patients from the pre-menopausal cohort were more frequently allocated to the higher-rated risk groups for both early and late recurrence and the highest estimated absolute chemotherapy benefit group. These findings, despite the small sample size of the study, are consistent with established knowledge that pre-menopausal women with ER+/HER2- often experience more aggressive disease, higher recurrence rates and lower survival [21]. Chemotherapy-induced amenorrhea via ovarian function suppression may be able to provide a significant survival benefit, a matter of extensive clinical research currently [6,21].

Last, but not least, the present study’s observations (albeit in small numbers) seem to be also in agreement with EPclin results from the UNIRAD (UNIversal RADiotherapy) trial [22], according to which the majority of node-positive patients with grade 3 and Ki-67 ≥20% were allocated to the high-risk EPclin subgroup. Furthermore, particularly for pre-menopausal patients, it is noteworthy that more than 20% of patients with node-positive disease had an EPclin score of ≥4.8. This threshold was the one assumed to define the benefit of a CDK4/6 inhibitor addition according to Penault-Llorca et al. [22].

Overall, our findings are in line with the previously published studies by Klein et al. [19] and Constantinidou et al. [5], which demonstrated that EndoPredict® can be performed both in pre- and post-menopausal ER-positive/HER2-negative early breast cancer patients and it has the ability to classify patients into either low or high risk of recurrence independently of their menopausal and nodal status and sometimes differently from what the standard clinicopathological features would impose [9,17,23].

Study limitations

This retrospective study was conducted on a cohort of 200 patients, leading to small subgroup sizes. The reports were consecutively selected for 100 pre-menopausal and 100 post-menopausal patients from a large group of ER-positive/HER2- breast-cancer patients who were tested in the Molecular Biology Laboratory of the Bioiatriki Group from April 20, 2026, backward. This study did not include the decision on any treatment protocol following the evaluation of the EndoPredict result, nor did it collect any patient follow-up information. Consequently, it presents an evaluation of predicted recurrence risks rather than any clinical outcomes of treatment decisions based on the EndoPredict result for patients. The study focused exclusively on the likelihood of distant recurrence within 0-10 and five to 15 years after five years of endocrine therapy alone and the absolute chemotherapy benefit. The study’s generalizability is therefore limited to these parameters and it is further restricted by the strictly descriptive statistical analysis performed.

## Conclusions

Our findings underscore the ability of EPclin Risk score to stratify ER+/HER2- breast cancer patients independently of their menopausal and nodal status and classify patients’ recurrence risk differently than traditional clinicopathological assessment, involving tumor size, nodal status, Ki-67 expression, and grade. EPclin also demonstrated the ability to identify a subgroup of low-risk patients among the pre-menopausal patients with node-positive disease. This feature can provide treating physicians with the option to reassess the use of chemotherapy in selected pre-menopausal breast cancer patients. However, the lack of follow-up data and clinical outcomes limits conclusions about its clinical value and potential for treatment de-escalation. Larger cohort studies in the future will be able to address these questions.
